# Multi‐omics insights into surface charge effects to decode the interplay of nanoplastics and bacterial antibiotic resistance

**DOI:** 10.1002/imt2.70056

**Published:** 2025-06-14

**Authors:** Houyu Li, Yinuo Ding, Yan Xu, Wei Liu

**Affiliations:** ^1^ Agro‐Environmental Protection Institute Ministry of Agriculture and Rural Affairs Tianjin China; ^2^ Université Claude Bernard Lyon 1, Laboratoire d' Ecologie Microbienne, UMR CNRS 5557, UMR INRAE 1418 Villeurbanne France; ^3^ Department F. A. Forel for Environmental and Aquatic Sciences, Section of Earth and Environmental Sciences and Institute for Environmental Sciences University of Geneva Geneva Switzerland

## Abstract

Multi‐omics approaches revealed how nanoplastics with different surface charges influence antibiotic resistance in *Escherichia coli* K12. Positively charged nanoplastics enhanced antibiotic resistance by upregulating genes and proteins linked to oxidative stress tolerance and efflux pumps, and promoted antibiotic resistance genes transfer via conjugation and transformation. In contrast, negatively charged nanoplastics disrupted biofilm formation and metabolism, potentially reducing antibiotic resistance. These findings highlight the critical role of nanoplastics' surface properties in shaping microbial resistance dynamics and highlight emerging risks posed by nanoplastics to public health through accelerated antibiotic resistance propagation.

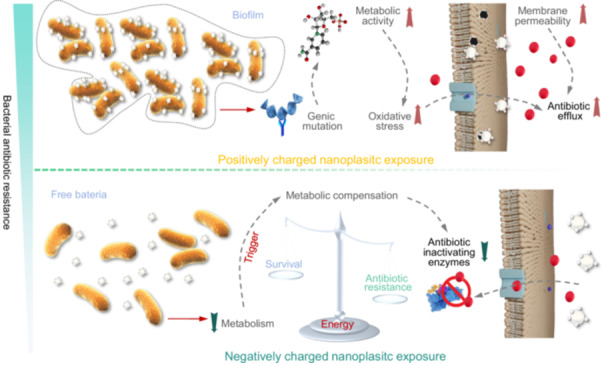

To the Editor,

Plastic pollution has become a global concern due to its persistence and ubiquity in aquatic and terrestrial ecosystems [1]. Environmental degradation of plastics results in microplastics (<5 mm) and nanoplastics (NPs, <1 μm), which can also be produced intentionally. Among these, NPs have attracted increasing attention for their potential to co‐occur with pollutants and influence bacterial antibiotic resistance, thereby posing a risk to environmental and public health [2].

Antibiotic resistance in bacteria arises from multiple factors, including antibiotic misuse, environmental contaminants, and horizontal gene transfer (HGT) [3]. NPs have been shown to affect bacterial metabolism and community structure, contributing to antibiotic resistance development. However, the effects of NP surface properties, particularly surface charge, on antibiotic resistance remain underexplored. Surface charge influences NP–cell interactions, membrane permeability, and gene expression, which may facilitate or hinder the spread of antibiotic resistance genes (ARGs).

NPs can carry various surface functional groups depending on their origin, whether derived from degradation or engineered directly [4]. Environmental conditions further alter NPs surface properties, complicating their biological effects [5]. For instance, amine‐functionalized NPs (PS‐NH₂) disrupt bacterial membranes more than carboxyl‐functionalized NPs, and their cellular uptake differs significantly across bacterial types [6]. However, few studies have investigated how these differences affect horizontal ARG transfer and the underlying molecular mechanism of NPs on the dissemination of AR. Thus, this study aims to address this knowledge gap by examining how positively and negatively charged polystyrene NPs influence antibiotic resistance evolution in *Escherichia coli* K12 at environmentally relevant concentrations. It will contribute to a better understanding of NP‐induced antibiotic resistance risks and inform environmental strategies for controlling ARG dissemination.

## RESULTS AND DISCUSSION

### Positively charged NPs enhanced bacterial antibiotic resistance

**FIGURE 1:**
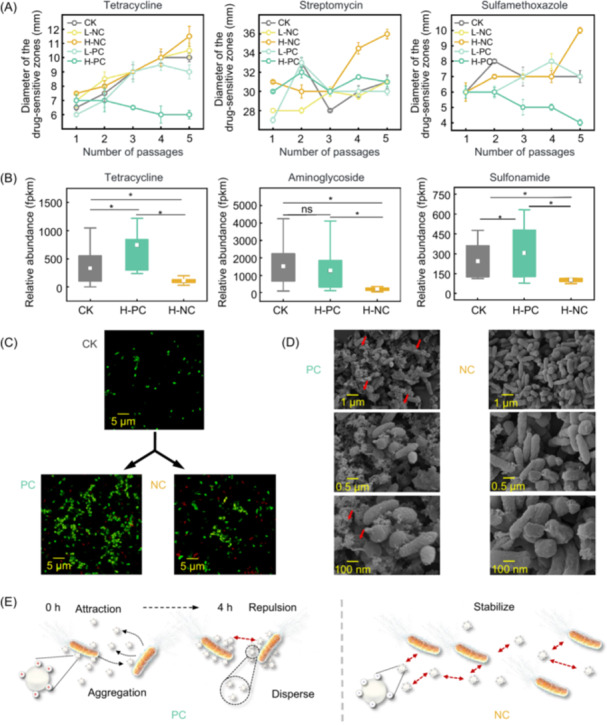
Variations of antibiotic resistance in *Escherichia coli* K12 (*E. coli* K12) and the interaction behavior of *E. coli* K12 with nanoplastics (NPs). (A) Drug sensitive test of *E. coli* K12 exposure to NPs with different charge under low (5 mg/L) or high (50 mg/L) concentration, which was exposed to culture after 5 passages continuously. Total of five treatments, including a control without NPs (CK), NPs with positive charge under low concentration (l‐PC) and high concentration (H‐PC), NPs with negative charge under low concentration (l‐NC) and high concentration (H‐NC). Drug‐sensitive test for tetracycline, Streptomycin, and Sulfamethoxazole antibiotics are shown here, and drug‐sensitive test for ampicillin, kanamycin, and norfloxacin antibiotics are provided in Figure S1. (B) Distribution of the relative abundance of antibiotic resistance genes (ARG) subtypes under different treatments, including tetracycline, sulfonamide, aminoglycoside, fluoroquinolones, and β‐lactam resistance. The tetracycline, aminoglycoside, and sulfonamide categories of ARGs are shown here, others are provided in Figure S2. The gray, green, and orange lines represent the CK, PC, and NC treatments, respectively. And the statistical analysis of differences between treatments was calculated using Mann–Whitney *U* test. “ns” indicates no significant difference, with *p* > 0.05. “*” represents a significant difference, with *p* < 0.05. (C) Aggregation behavior of NPs and *E. coli* in LB, and their interactions are observed by laser scanning confocal microscope. The red dot represents NPs and green dot represents *E. coli*. The cells are densely packed in positively charged (PC) NPs treatment, which limits the visible migration distance. While in negatively charged (NC) NPs treatment, the cell proximity is more apparent due to the increased intercellular space. (D) Scanning electron microscope (SEM) image displays the adsorption of NPs on the cell. (E) The schematic diagram shows the interaction of NPs carrying different charges with *E. coli*. The left image represents the dynamic process of the interaction between NPs with positively charged and *E. coli*, and right image represents the NPs with negatively charged and *E. coli*.

To evaluate how surface‐charged NPs affect bacterial antibiotic resistance, *E. coli* K12 was exposed over 540 generations to positively and negatively charged NPs. Drug sensitivity assays revealed that low NP concentrations (5 mg/L) did not significantly impact inhibition zone diameters compared to the control (CK). However, at 50 mg/L, substantial changes were observed (Figure 1A and Figure S1). In the high concentration of positively charged NPs (H‐PC) treatment, inhibition zones for sulfamethoxazole, tetracycline, and norfloxacin decreased by ~1 to 14 mm, indicating increased resistance. In contrast, negatively charged NPs (H‐NC) treatment with high concentration significantly enlarged the zones (e.g., 1.5 mm for tetracycline, 5 mm for streptomycin) (Mann–Whitney *U* test, *p* < 0.05), suggesting suppressed resistance.

The relative abundance of ARGs was further quantified in *E. coli* K12. Positively charged NPs (PC‐NPs) notably increased ARGs levels, while negatively charged NPs led to reductions (Figure 1B and Figure S2). For example, tetracycline, fluoroquinolones, and sulfonamide resistance genes in H‐PC increased, ranging from 13.89 to 2760.65, 3.08 to 2176.44, and 186.11 to 388.42 fpkm, respectively, while the H‐NC treatment reduced the abundance of ARGs, ranging from 98.77 to 3846.50 fpkm. Specific genes like *tet*(42), *emr*B, and *sul*2 were markedly elevated in H‐PC (Figure S3), correlating with mechanisms such as target protection and efflux [7, 8, 9]. Meanwhile, negatively charged NPs (NC‐NPs) likely reduced resistance via antibiotic inactivation, as seen in decreased expression of resistant to oxyimino cephalosporins Beta‐lactamase‐8 (*ROB*‐8), oxacillinase‐9 (*OXA*‐9), and cephamycinase‐45 (*CMY*‐45) than CK. Thus, the mechanisms of NPs with different surface charges on antibiotic resistance in *E. coli*. K12 were investigated under high concentration (50 mg/L) below.

### Interaction between NPs with different surface charges and *E. coli*

The behavior of NPs in culture medium and their interactions with bacteria play a critical role in the evolution of bacterial antibiotic resistance. As shown in Figure S4A,B, both PC‐ and NC‐NPs were unstable upon dispersion, with stronger aggregation in the PC treatment (224.91 ± 57.31 nm) than in the NC treatment (123.92 ± 47.61 nm). This may result from organic macromolecules in Luria‐Bertani medium forming a “bio‐corona” on NP surfaces, increasing surface roughness and attraction, thus promoting aggregation. This aggregation increases local NPs concentration and bacterial stress, potentially enhancing resistance evolution, especially with PC‐NPs.

Over time, the NP size in the PC treatment decreased to 100.84 ± 11.23 nm, while NC‐NPs stabilized at 150 ± 24.52 nm, indicating lower stability of PC‐NPs. This instability may be due to a “bridging effect” where PC‐NPs bind to negatively charged *E. coli* surfaces, decreasing zeta potential and reducing aggregate size (Figure S4C,D). Zeta potentials were similar (NC: 16.06 ± 0.59 mV; PC: 14.92 ± 0.53 mV), but NC‐NPs were more electrostatically repelled, limiting bacterial attachment and biofilm formation. Without biofilm protection, bacteria were more antibiotic‐sensitive and expressed fewer ARGs, especially those for antibiotic‐inactivating enzymes.

Laser scanning confocal microscopy and scanning electron microscopy (SEM) observation (Figure 1C,D) confirmed NPs aggregation and membrane damage in PC treatments, suggesting enhanced antibiotic permeability and efflux. SEM imaging further supported this hypothesis, showing that the surface of *E. coli* was not encapsulated by biofilm under NC‐NPs exposure. Conversely, the aggregated NC‐NPs formed a physical barrier between the bacteria, potentially preventing intercellular “communication” (Figure 1E), which likely reduced HGT or the transformation of ARGs [10]. Differently, the mirrored effect of the PC‐NPs treatment resulted in a larger aggregation of *E. coli* K12 than other treatments, suggesting that the PC‐NPs exposure could enhance microbial aggregation, which may contribute to the ARGs proliferation. This aggregation could significantly facilitate bacteria communication, thereby promoting the horizontal transfer of ARGs.

### Transcriptomic and proteomics insights into antimicrobial resistance triggered by different charged NPs

**FIGURE 2:**
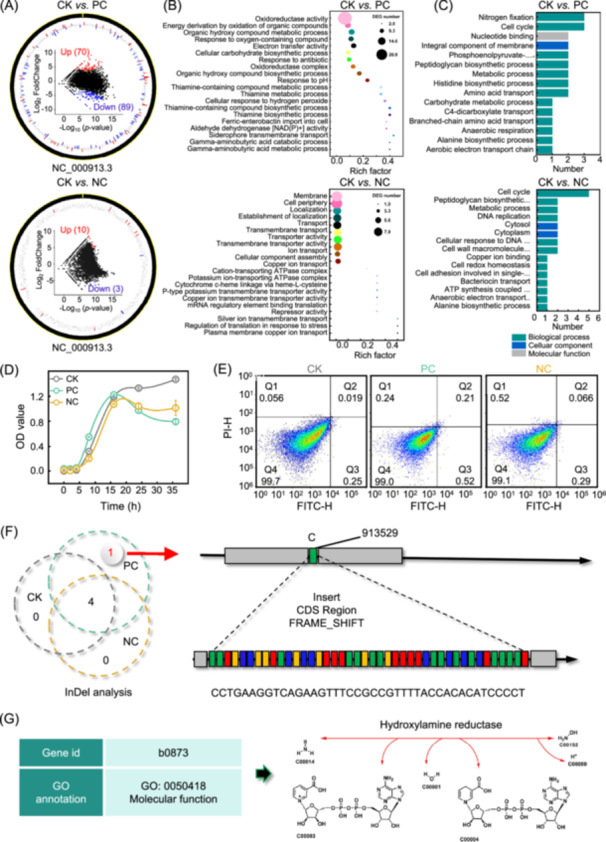
The underlying mechanisms of nanoplastics (NPs) with different surface charges on the bacterial antibiotic resistance evolution. (A) The volcano plots are performed to screen for the differential expression genes (DEGs) (|FoldChange| > 5, Mann–Whitney *U* test, *p* < 0.05) in the PC and NC treatments compared with CK treatment. The red dot represents the upregulation genes, and the blue dot represents the downregulation genes. (B) Enrichment analysis of DEGs based on the Gene Ontology (GO) database under different treatments. The size of points represents the number of DEGs. (C) Enrichment analysis of differential expression proteins (DEPs) based on the GO database. (D) The growth curves of *E. coli* under different treatments. (E) Flow cytograms show the proportion of apoptotic individuals in the strains under different treatments. Q1 (Upper left): Cell debris that has lost its cell membrane, or dead cells caused by other reasons; Q2 (Upper right): Late apoptotic; Q3 (Lower right): Early apoptotic; Q4: Live cells (Lower left). The proportion of apoptotic individual = Q2 + Q3. FITC‐H, Fluorescein Isothiocyanate ‐ Height; PI‐H, Propidium Iodide‐Height. (F) The difference analysis of mutation detection in *E. coli* under different treatments. (G) The annotation of differential variant sites. NC, negatively charged; PC, positively charged.

We used transcriptomics to explore how NPs with different surface charges influence bacterial antibiotic resistance. Compared to CK, PC‐NPs exposure resulted in 70 upregulated and 89 downregulated differentially expressed genes (DEGs), significantly more than the NC‐NPs treatment (10 up, 3 down; Mann–Whitney *U* test, *p* < 0.05) (Figure 2A). Notably, a significant correlation (Permutation test, *p* < 0.05, Spearman |*r*_*s*_| > 0.6) was observed between the expression of these DGEs and the variation in ARGs (Figure S5), indicating that surface‐charged NPs alter *E. coli* K12 physiology, more strongly under PC‐NPs exposure. Gene Ontology (GO) analysis revealed that oxidoreductase activity was the main function associated with DEGs in PC‐NPs treatment (Figure 2B). A significant positive relationship was observed between DEGs related to oxidoreductase activity and ARGs (Mann–Whitney *U* test, *p* < 0.05) (Figure S6). Genes encoding flavohemoprotein (*hmp)*, thiamine biosynthesis protein (*thiH)*, thiamine biosynthesis protein (*thiC)*, and glycosyltransferase family protein D *(gfcD)* were upregulated 1.28, 1.35, 1.60, and 2.58 times in the PC‐NPs treatment compared with CK treatment (Figure S7), indicating enhanced resistance to oxidative stress and supporting survival under antibiotic pressure. Increased oxidoreductase activity may also boost efflux pump function, aiding antibiotic resistance by promoting efflux and reducing permeability [11]. Additional DEGs involved in energy metabolism and responses to oxygen compounds (e.g., *ycjO*, *ycjP*) were upregulated up to 15.68 times in PC‐NPs treatment. These contribute to metabolic reprogramming under stress, which can further drive resistance evolution. DEGs also impacted cell membrane functions, such as biofilm formation [12]. Genes encoding potassium‐transporting ATPase subunit A (*kdpA*), reductive chlorate resistance regulator (*rclR*), surface exclusion protein D (*sfmD*), and copper/silver efflux system protein (*cusA*) were also significantly upregulated up to 14.88 times (Figure S7), suggesting disrupted biofilm formation (Mann–Whitney *U* test, *p* < 0.05). For example, *rclR* downregulated quorum sensing [13], whereas *sfmD* altered membrane synthesis [14], thereby weakening bacterial adhesion and reducing biofilm‐associated resistance, especially in the presence of NC‐NPs exposure.

Proteomics analysis confirmed these findings. PC‐NPs treatment induced 82 differentially expressed proteins (DEPs), while NC‐NPs caused 51 (Mann‐Whitney *U* test, *p* < 0.05). PC‐NPs led to 49 up‐ and 33 downregulated proteins, while NC‐NPs had 28 up‐ and 23 downregulated (Figure S8A,B). Consistent with the transcriptome analysis, a significant correlation between DEPs and DEGs was determined by Spearman's correlation analysis (*r*_*s*_ = 0.769, *p* = 0.0017). These results reinforced the idea that exposure to PC‐NPs had a more significant impact on bacteria cellular functions, thereby promoting the evolution of antibiotic resistance (Figure S8C). GO‐based functional annotations showed enrichment in nitrogen fixation, cell cycle, and nucleotide binding under PC‐NPs, with Log_2_FoldChange increases from 2.03 to 3.21 (Figure 2C). These results are consistent with transcriptomic data suggesting enhanced metabolic and antioxidant defenses that contribute to the efficiency and resistance of efflux pumps [15].

In contrast, DEPs under NC‐NPs treatment were mainly linked to the cell cycle and peptidoglycan biosynthesis, exhibiting a downregulation of 2.24–3.43 in Log_2_FoldChange. Inhibited peptidoglycan synthesis imposes metabolic stress and slows growth, thus inhibiting biofilm formation. The suppression of peptidoglycan biosynthesis induces metabolic stress and resource constraints in bacteria, driving them to undergo adaptive evolution [16]. These stresses reduce the capacity to express antibiotic‐inactivating enzymes to sustain essential life activities under environmental stresses, explaining the suppressed ARGs expression in NC‐NPs treatment. These results suggest that PC‐NPs promote bacterial antibiotic resistance by enhancing efflux, decreasing cell membrane permeability, and metabolic adaptation, whereas NC‐NPs limit resistance by disrupting biofilm formation and weakening antibiotic inactivation pathways.

### Genetic recombination plasmid replication of *E. coli* was triggered by PC‐NPs

Bacterial growth curves reflect metabolic activity and density, with faster growth indicating higher activity. As shown in Figure 2D, *E. coli* K12 in PC‐NPs and CK treatments grew more rapidly in the early phase than in NC‐NPs. PC‐NPs showed the highest initial growth rate, suggesting enhanced metabolic activity. However, during the logarithmic phase, biomass in PC‐NPs declined sharply, resulting in the lowest cell density during the stationary phase (CK > NC‐NPs > PC‐NPs). This decline likely resulted from increased apoptosis caused by membrane rupture induced by PC‐NPs. In contrast, NC‐NPs mainly suppressed metabolism, causing limited apoptosis and slower initial growth, but ultimately leading to higher biomass in the stationary phase.

Flow cytometry analysis confirmed higher apoptosis rates in PC‐NPs (0.73%) compared to NC‐NPs (0.36%) and CK (0.28%) (Figure 2E). PC‐NPs may also increase DNA replication errors or impair repair mechanisms, promoting adaptive mutations and resistance [17]. To ascertain whether mutations occurred in *E. coli* K12 after exposure to NPs with different charges, re‐sequencing was performed to compare the sequences of *E. coli* K12 with Reference Genome (GCF_000005845.2). Genome re‐sequencing revealed no single‐nucleotide polymorphism differences across treatments but detected an indel variation in the PC‐NPs treatment at position 913529 in a coding region (Figure 2F). Thereafter, the function of the variant site was annotated (Figure 2G), revealing its association with hydroxylamine reductase (Hcp) (GO: 0050418, b0873) [18]. Hcp, an enzyme involved in nitrate/nitrite reduction and oxidative stress response, may reduce intracellular oxidative stress and aid antibiotic resistance [19], supporting transcriptomic and proteomic findings.

NPs exposure also influenced plasmid dynamics. The whole genome sequencing showed that only one contig was annotated as a plasmid in CK and NC‐NPs treatments, but two in PC‐NPs (Figure S9). Plasmid length increased by 15,245 bp in PC‐NPs, suggesting promoted plasmid replication or enhanced the acquisition of free plasmid, potentially promoting vertical transmission of ARGs. Conversely, plasmid length decreased by 33,220 bp in NC‐NPs, indicating plasmid loss, possibly due to reduced cell cycling and metabolic activity. Slower division limits plasmid replication, and under stress, bacteria may reduce plasmid burden for metabolic compensation [20]. These results illustrate a “division of labor” principle in bacterial responses: under PC‐NPs, bacteria invest more energy in resistance and adaptation, while under NC‐NPs, they prioritize survival over energy‐intensive resistance mechanisms, contributing to reduced ARGs abundance.

### HGT in *E. coil* was enhanced by NPs with positive surface charges

To assess whether NPs enhance HGT of ARGs in *E. coli* K12, we developed conjugation and transformation models. Results showed that HGT capacity followed the order PC‐NPs > NC‐NPs ≈ CK, with conjugation frequencies of 2.36%, 0.64%, and 0.54%, respectively (Figure S10A). Transformation efficiency also increased, with PC‐NPs exposure resulting in a 3.75‐times rise, and NC‐NPs 1.5 times rise, compared to CK (Figure S10B). Swarming tests revealed greater motility in PC‐NPs‐treated bacteria, likely due to electrostatic interactions enhancing mobility (Figure S10C), thus potentially increasing intercellular contact and ARGs transfer.

To evaluate these effects in a complex microbial community, we quantified plasmid transfer from *E. coli* to environmental bacteria (Figure S10D). PC‐NPs exposure significantly increased conjugator abundance (0.71%) and transfer frequency (0.0107), compared to CK (0.46%, 0.0061) and NC‐NPs (0.45%, 0.0066) (Mann–Whitney *U* test, *p* < 0.05). These findings demonstrate that PC‐NPs promote ARGs dissemination via enhanced HGT in *E. coli*, raising concerns about increased resistance spread when such particles interact with environmental microbes.

## CONCLUSION

This study found that high concentrations (50 mg/L) of PC‐NPs significantly increased antibiotic resistance in *E. coli* K12 by enhancing oxidative stress tolerance and antibiotic efflux pump activity. In contrast, NC‐NPs inhibited resistance by disrupting biofilm formation and metabolism, potentially forcing bacteria to lose resistance as a survival strategy. Notably, PC‐NPs also promoted both vertical transmission and HGT of ARGs, escalating the risk to human health. Future research should investigate a broader range of NPs to better assess their effects on antibiotic resistance, and elucidate the dynamics of bio‐corona formation, surface property alterations, and their underlying mechanisms in modulating antibiotic resistance.

## METHODS

Detailed experimental materials and procedures, including sample collection and processing techniques, and statistical analysis, are provided in the Supplementary Material (Figures S11 and S12, Methods).

## AUTHOR CONTRIBUTIONS

**Houyu Li**: Writing—original draft; data curation; formal analysis; investigation; validation; visualization; methodology. **Yinuo Ding**: Visualization; methodology; data curation; formal analysis. **Yan Xu**: Investigation; validation; writing—review and editing; project administration; conceptualization; funding acquisition. **Wei Liu**: Writing—review and editing; investigation; validation; resources; supervision; conceptualization.

## CONFLICT OF INTEREST STATEMENT

The authors declare no conflicts of interest.

## ETHICS STATEMENT

No animals or humans were involved in this study.

## Supporting information


**Figure S1:** Drug sensitive test of *E. coli* K12 exposure to NPs with different charge under low (5 mg/L) or high (50 mg/L) concentration.
**Figure S2:** Distribution of the relative abundance of Fluoroquinolones and β‐lactam ARGs under different treatments.
**Figure S3:** Heatmap shows that the relative abundance of ARG subtypes under different treatments.
**Figure S4:** Z‐average hydrodynamic diameter and ζ‐Potential of NPs in Luria‐Bertani.
**Figure S5:** Procrustes analysis displays the relationships between DEGs and ARGs under different treatments.
**Figure S6:** Heatmap shows the relationships between DEGs and ARGs.
**Figure S7:** Expression of DEGs under different treatments.
**Figure S8:** Proteomic analysis of *E. coli* under exposure to NPs with different surface charges.
**Figure S9:** Whole genome sequencing (WGS) and the number and length of plasmid carried by the *E. coli*.
**Figure S10:** Horizontal gene transfer ability under different treatments.
**Figure S11:** Evironmental design diagram.
**Figure S12:** Morphology of NPs (20 nm) is examined using SEM.

## Data Availability

The data that support the findings of this study are available from the corresponding author upon reasonable request. All the sequencing data have been deposited in NCBI under BioProject accession number PRJNA1266484 (https://www.ncbi.nlm.nih.gov/bioproject/PRJNA1266484). The data and scripts used are saved in GitHub https://github.com/lhu1028/Imeta/tree/master. Supplementary materials (methods, figures, graphical abstract, slides, videos, Chinese translated version, and update materials) may be found in the online DOI or iMeta Science http://www.imeta.science/.
