# Supplementary material for: Multi‐omics insights into surface charge effects to decode the interplay of nanoplastics and bacterial antibiotic resistance
Source: Imeta. 2025 Jun 14;4(4):e70056. doi: 10.1002/imt2.70056 (PMC12371250; doi:10.1002/imt2.70056)
Supplement: Supplementary file 1 — Figure S1: Drug sensitive test of E. coli K12 exposure to NPs with different charge under low (5 mg/L) or high (50 mg/L) concentration. Figure S2: Distribution of the relative abundance of Fluoroquinolones and β‐lactam ARGs under different treatments. Figure S3: Heatmap shows that the relative abundance of ARG subtypes under different treatments. Figure S4: Z‐average hydrodynamic diameter and ζ‐Potential of NPs in Luria‐Bertani. Figure S5: Procrustes analysis displays the relationships between DEGs and ARGs under different treatments. Figure S6: Heatmap shows the relationships between DEGs and ARGs. Figure S7: Expression of DEGs under different treatments. Figure S8: Proteomic analysis of E. coli under exposure to NPs with different surface charges. Figure S9: Whole genome sequencing (WGS) and the number and length of plasmid carried by the E. coli. Figure S10: Horizontal gene transfer ability under different treatments. Figure S11: Evironmental design diagram. Figure S12: Morphology of NPs (20 nm) is examined using SEM. [file IMT2-4-e70056-s001.docx]

**Supporting information to**

**Multi-Omics Insights into Surface Charge Effects to Decode the Interplay of Nanoplastics and Bacterial Antibiotic Resistance**

**Running title**: Surface Charge Effects of Nanoplastics on the Bacterial Antibiotic Resistance

Houyu Li^1^, Yinuo Ding^1^, Yan Xu^1*^, Wei Liu ^2, 3*^

^1^Agro-Environmental Protection Institute, Ministry of Agriculture and Rural Affairs, Tianjin 300191, China

^2^Université Claude Bernard Lyon 1, Laboratoire d’ Ecologie Microbienne, UMR CNRS 5557, UMR INRAE 1418, Vet Agro Sup, Villeurbanne 69622, France

^3^Department F. A. Forel for Environmental and Aquatic Sciences, Section of Earth and Environmental Sciences and Institute for Environmental Sciences, University of Geneva, Geneva 1205, Switzerland

* Correspondence: [xuyan@caas.cn](mailto:xuyan@caas.cn) (Yan Xu), [wei.liu@univ-lyon1.fr](mailto:wei.liu@univ-lyon1.fr) (Wei Liu)

**METHODS**

**Experimental design and sample collection**

The detailed process of the experimental design for this study was displayed in Figure S11. Herein, the hydroxylated polystyrene nanospheres suspension (4% w/v, 20 nm) (XFNANO, China) with -NH_2_ and -COOH functional group, representing positive (PC) and negative (NC) surface charge, respectively, were selected to expose to *E. coli* K12. The concentrations of nanoplastics (NPs) were set to be environmentally relevant, which were at a concentration of 5 mg/L (Low concentration) and 50 mg/L (High concentration). Overall, five treatments were conducted: a control group without NPs (CK), NPs with positive charge (PC) at low (L-PC) and high (H-PC) concentrations, and NPs with negative charge (NC) at low (L-NC) and high (H-NC) concentrations. Each treatment was replicated three times. Moreover, to ensure that NPs were homogeneously dispersed, an ultrasonic cleaner (KQ5200DE, Jiangsu) was employed to shock the NPs for 5 s prior to experimental application. And the dispersion of NPs was re-characterized before undertaking the experiments, and provided it in Figure S12. The morphology of NPs (20 nm) was examined using scanning electron microscopy (SEM; JEOL JSM-7001FA, Tokyo, Japan). Samples were mounted on aluminum stubs covered with 5 × 5 mm silica mirrors (Agar Scientific, G3390). NPs were suspended in ultrapure water, and 5 μL of the suspension was deposited onto the holders, air-dried, and then sputter-coated with a 5 nm layer of gold.

Throughout the experiment, *E. coli* K12 was cultured in Luria-Bertani (LB) liquid medium, which was refreshed every 48 h to provide the necessary nutrients for bacterial growth. Simultaneously, the NPs were also added with each medium update, as described in the previous study [1]. And the strains were continuously incubated for 540 generations (5 passages) under different treatments. After continuous cultivation with NPs exposure, the *E. coli* fluids, which were cultured to a concentration of 10^6^ CUF/mL, were collected to subsequent drug susceptibility test and multi-omics analysis.

**Drug susceptibility test**

Drug susceptibility tests were used to examine the susceptibility of *E. coli* K12 to six commonly used antibiotics under NPs exposure. The antibiotics tested included kamycine, norfloxacin, tetracycline, ampicillin, streptomycin, and sulfamethoxazole. These tests were conducted on LB solid medium inoculated with strains from each passaging culture period under different treatments. Meanwhile, the tablets of different antibiotic medicines were adhered to medium and placed them in an incubator at 37 °C for the 24 h of cultivation. We measured the inhibition zones around antibiotic tablets to assess *E. coli* K12 susceptibility under different treatments, following established protocols of the previous works [2].

**RNA extraction and transcriptions analysis of *E. coil***

The Invitrogen TRIzol (Thermo Fisher, USA) was used to extract ribonucleic acid (RNA) from *E. coli* K12 before each passaging in different treatments*.* Total RNA was extracted using the Trizol method, which involves several key steps: First, 1 mL of Trizol reagent was added per 50–100 mg of tissue or 5–10 × 10^6^ cells, and the sample was thoroughly homogenized or lysed to ensure complete dissociation of the nucleoprotein complex. After homogenization, the sample was incubated at room temperature for 5 min, followed by the addition of 0.2 mL of chloroform per 1 mL of Trizol. The sample tube was securely capped and vigorously shaken by hand for 15 s, then incubated at room temperature for 2–3 min before centrifugation at no less than 12,000 g for 15 min at 4 ℃. This process resulted in the separation of the mixture into a lower phenol-chloroform phase, an interphase, and a colorless upper aqueous phase containing the RNA. The aqueous phase was transferred to a fresh tube, avoiding disturbance of the interphase, and RNA was precipitated by adding 0.5 mL of isopropanol per 1 mL of Trizol, followed by incubation at room temperature for 10 min and centrifugation at no less than 12,000 g for 10 min at 4 ℃ forming an RNA pellet at the bottom of the tube. The supernatant was removed, and the RNA pellet was washed once with 75% ethanol, using at least 1 mL per 1 mL of Trizol, mixed by vortexing, and centrifuged at no less than 7500 g for 5 min at 4 ℃. The RNA pellet was briefly air-dried for 5–10 min, ensuring it did not completely dry, and then redissolved in RNase-free water by pipetting up and down or incubating the tube at 55–60 ℃ for 10–15 min, with the volume of water adjusted to achieve the desired RNA concentration. And the RNA purity was detected by Nanodrop 2000 (Implen, CA, USA) and the length of RNA fragments was examined by Agilent 2100 (Agilent Technologies, CA, USA). After the samples were of satisfactory quality, the library was constructed by the strand-specific library construction method, and the insert size of the library was detected using an Agilent 2100, which the effective concentration of the library was > 4 nM. Then, the library was pooled and selected for on-line measurement using Illumina high-throughput sequencing platform (HiSeqTM2500/4000). To ensure the effectiveness of the subsequent analysis of sequencing data, the raw data were processed using specific filtering criteria as follows: (1) Filtering of Reads with sequencing adapters, (2) Filtering of reads with a proportion of N (uncertain base) content greater than 1%, and (3) Filtering of Reads with a content of low-quality bases (Q ≤ 20) greater than 50%. Finally, the data were analyzed and compared using Bowtie2 (v2.3.5.1).

**DNA Extraction and Quantification of ARGs**

Genomic deoxyribonucleic acid (DNA) was extracted using a Bacterial genomic DNA extraction kit (DP302-02, TIANGEN). The concentration and quality of the total genomic DNA were determined using a NanoDrop2000 Spectrophotometer (Thermo Fisher Scientific). DNA library (350 bp) for Illumina/BGI sequencing was constructed for each accession according to the manufacturer’s specifications. Then, sequencing was performed on an Illumina HiSeq XTen/NovaSeq/BGI platform (Biomarker Technologies, Beijing, China). The raw reads were filtered based on the following criteria: (1) pair-end reads with > 10% ‘N’ bases and (2) Reads, on which more than 50% of the bases have a quality score less than 20. Finally, high-quality sequences were obtained for subsequent analyses.

Further, the sample genomic DNA was employed for Polymerase Chain Reaction (PCR) amplification to form a sequencing library to obtain raw reads, which was performed via the Next-generation sequencing. Subsequently, the raw reads were screened out to obtain clean reads based on the data quality control process. Those clean reads were compared with the comprehensive antibiotic resistance (CARD) database to finally obtain the information of antibiotic resistance genes (ARGs).

**Reseq-ONT (20K)**

Experimental procedures for Reseq-ONT (20K) were performed according to standard protocols which were provided by Oxford Nanopore Technologies (ONT) [3]. Raw data were obtained based on the Nanopore sequencing platform, and the fast5 format data were converted to fastq format by Guppy (v3.2.6) software in the MinKNOW package for subsequent analyses. Further, low quality and short fragment reads were filtered based on the filtering condition of length < 2000 bp. The filtered reads were then assembled using Canu (v1.5) software and the assembled results were corrected by the Racon (v3.4.3) based software. Meanwhile, the ringing and adjustment of the starting site was carried out by Circlator (v1.5.5) software, and the error correction was further performed by Pilon (v1.22) software with second generation data, eventually getting the genome with high accuracy.

**Protein extraction and liquid chromatography-tandem mass spectrometry analysis for Proteomics**

The samples were pulverized while under the influence of liquid nitrogen and transferred to a sterile centrifuge tube to lyse using sonication. Then, the total protein concentration was measured using Bicinchoninic Acid (BCA) protein quantification assay. Finally, the proteomics of strains with NPs exposure were tested by the LC-MS/MS analysis. The samples were separated using NanoElute system with a nanoliter flow rate, incorporating an analytical reversed-phase C18 column (IonOpticks, Australia, 25 cm × 75 µm, C18 packing 1.6 µm). Detailed information for NanoElute system as followed [4]: The mobile phases comprised of 0.1 % formic acid aqueous solution in phase A and 0.1% formic acid acetonitrile aqueous solution (with acetonitrile at 100 %) in phase B. The temperature of the analytical column was controlled by an integrated column temperature box at 50 ℃. The gradient was maintained for 60 min. The temperature of the analytical column was controlled by an integrated column oven at 50 ℃. The sample volume was maintained at 200 ng, with a flow rate of 300 nL/ min, and the gradient spanned over 60 min. The 60 min liquid-phase gradient over this period was as follows: during 0–45 min, the B-liquid went from 2%–22%; during 45–50 min, the B-liquid linear gradient increased from 22% to 35%; during 50–55 min, the B-liquid linear gradient increased from 35% to 80%; and during 55–60 min, liquid B was maintained at 80%. Subsequently, the samples underwent chromatography, followed by Mass Spectrometry (MS) data acquisition using a Trapped Ion Mobility Spectrometry (TIMs) Time of Flight (TOF) Pro mass spectrometer in the Data-Dependent Acquisition (DDA) Parallel Accumulation Serial Fragmentation (PASEF) mode. The raw data from MS was analyzed using FragPipe (uniprot_xiwashi_jun_20221101.fasta database), incorporating decoys and contaminants libraries to manage for false-positive rates from random matching, as well as negate the impact of contaminating proteins.

**The behavioral characteristics of NPs with different surface charges**

The change of size and zeta potential under treatments with NPs carrying different charges, which represent the aggregation behavior of NPs, were detected using the Zetasizer Nano (Malvern, Nano-ZS) at the 0, 0.5, 2, and 4 h, respectively. Subsequently, the aggregation behavior of NPs in the LB medium was visualized by the laser scanning confocal microscope (A1R HD25, Nikon, Japan). To enable visualization of the interaction between NPs and *E. coli* K12, carboxyl and amine fluorescence NPs (CD Bioparticals, 1% w/v, 20 nm, λexi, 460 nm, λemi, 500 nm) were introduced into LB medium with *E. coli* K12 tagged with a red fluorescent marker, at a concentration of 10^6^ CUF/mL.

**Swarming test**

The swarming of the strains was viewed to display the motor activity of strains after continuous exposure with NPs carrying the different surface charge. The strains were inoculated in the center of the semi-solid medium, and incubated in the incubator at a constant temperature of 37 °C. And the spread diameter of the strains was observed at 24, 48, and 72 h, respectively.

**Conjugation transfer and transformation assays**

The conjugation transfer experiences were constructed to probe the conjugative transfer frequency in strains under different treatments. The strain *E. coli* K12, harboring the plasmid RP4 carrying ampicillin, kanamycin, and tetracycline resistance genes (ApR, KmR, and TcR) but no StrR was used as the donor. The HB101 carrying StrR (in the genome) were used as the recipients and lacked ApR, KmR, and TcR. They were placed together to mate for 12 h at 30 ^◦^C. After that, the number of transconjugants (ApR, KmR, TcR, and StrR) (NT) were counted and the results were presented as colony forming units per milliliter culture (cfu/mL). The number of recipients (StrR) (NS) was determined by culturing the bacteria on LB agar plates containing 30 mg/L of streptomycin (cfu/mL). Transfer frequency was also calculated using the formula:

Transfer frequency = NT (cfu/mL) / NS (cfu/mL)

Furthermore, the conjugation transfer experiment in the complex multi-microbial system also selected the strain *E. coli* K12 as the donor, and the microorganisms from the soil were used as receptors. And they were blended and co-cultured to mate for 12 h at 30 ^◦^C. Finally, the transconjugants were sorted by the flow cytometry method (INVI–TROGEN, USA).

Transformation assays were performed to evaluate whether exposure to NPs increased the transformation of free plasmids by *E. coli* K12. The RP4 plasmid was used as the model plasmid, with *HB101* selected as receptor. Detailed methods were referred from Wang et al. (2016) [5].

**Measurement of growth activity of bacteria**

The growth curves were performed to quantify the population density of the strains, which were plotted based on the optical density (OD) value. Further, the flow cytometer (INVITROGEN, USA) was used to determine the proportion of strain with apoptosis under different treatments. Before that, the strains should be stained by Annexin V-FITC/PI Apoptosis Detection Kit (GK3603-50T, GENVIEW). Detailed staining procedures were listed below: 1) Seed logarithmically growing cells in 6-well plates or 6 cm culture dishes, and ensuring a final collected cell number greater than 5 × 10^5^. 2) Treat cells according to experimental needs. At the time of collection, aspirate the culture medium into a centrifuge tube, wash the cells once with phosphate buffered saline (PBS), then digest the cells with trypsin without ethylene diamine tetraacetic acid (EDTA), and collect in a centrifuge tube (suspended cells can be directly collected by centrifugation). Note: After trypsin digestion, allow cells to recover for about 30 min in optimal cell culture conditions and medium before staining. Trypsin digestion temporarily disrupts the plasma membrane, allowing Annexin V to bind to phosphatidylserine on the cytoplasmic surface of the cell membrane, leading to false positive staining. 3) Mix the collected culture medium and cells thoroughly, centrifuge at 300 g for 5 min at 4 ℃, and discard the supernatant. Wash the cells with precooled PBS twice, each time centrifuging at 300 g for 5 min at 4 ℃. 4) Add 1× Binding Buffer to resuspend the cells to the same concentration (generally 1 × 10^6^ mL). 5) Take 1–2 × 10^5^ cells in suspension into a flow cytometry tube, add an appropriate amount of fluorescently labeled Annexin V and propidium iodide (PI), and gently mix. 6) Incubate at room temperature in the dark for 15–20 min. 7) Add 300 µL PBS to resuspend the cells, and proceed to analyze with a flow cytometer as soon as possible (within 1 h).

**SNP/INDEL calling**

Genomic DNA was extracted using a cetyltrimethylam-monium bromide (CTAB)-based protocol. The concentration and quality of the total genomic DNA were determined using a NanoDrop2000 Spectrophotometer (Thermo Fisher Scientific). DNA libraries (350 bp) for Illumina/BGI sequencing were constructed for each accession according to the manufacturer’s specifications. After DNA library construction, sequencing was performed on an Illumina HiSeq XTen/NovaSeq/BGI platform by a commercial service (Biomarker Technologies, Beijing, China), with 150-bp read lengths. Then, raw data (raw reads) of fastq format were initially processed through fastp software. In this step, clean data (clean reads) were obtained by filtering out adapter sequences, reads containing ploy-N and low-quality reads from raw data. Simultaneously, Q20, Q30, GC-content, and sequence duplication level of the clean data were calculated. All the downstream analyses were based on clean data with high-quality. Upon completion of the above steps, the adaptor sequences and low-quality sequence reads were removed from the data sets. Raw sequences were transformed into clean reads after data processing. These clean reads were then mapped to the reference genome sequence. bwa-mem2 software was used to map with GCF_000005845.2_ASM584v2 reference genome. Then, the mapping results were sorted, and duplicate reads were de-redundancy using samtools (v1.9). Furthermore, the Single Nucleotide Polymorphism (SNP) and Insertion/Deletion (INDEL) were called using the Haplotype Caller module in Genome Analysis Toolkit (GATK) (v3.8), and were filtered with the following parameters: Quality by Depth (QD) < 2.0 || Mapping Quality (MQ) < 40.0 || Fisher Strand Bias (FS) > 60.0 || Quality Score (QUAL) < 30.0 || Mapping Quality Rank Sum Test (MQrankSum) < -12.5 || Read Position Rank Sum Test < -8.0 –cluster Size 2 –cluster Window Size 5. Finally, SNP and InDels annotation was performed based on the reference genome using the SNP Effect Predictor (snpEff) software (v3.6c (build 2014-05-20)). SNPs were categorized into intergenic regions, upstream or downstream regions, and exons or introns. InDels in exons were grouped according to whether they led to a frame shift. Gene functions of SNPs and InDels were obtained by sequence comparison based on GO (Gene Ontology) database.

**Statistics and analysis**

Line graphs were drawn with Origin (2022, USA OriginLab), which displayed the change in antibiotic resistance phenotype. Also, the change of zeta potential under treatments with NPs carrying different charges were also displayed via the line graphs. Heatmaps were built via TB tool (v2.003) to demonstrate the abundance of ARGs under different treatments. And the statistical analysis of differences between treatments was calculated using Mann-Whitney *U* test, due to the data do not fit the normal distribution. It was performed by the function of wilcox.test() in the R language (v4.3.1), and *p*-values were corrected based on the Benjamini-Hochberg (BH) method. The volcano map was mapped based on the “Enhanced Volcano” package, which can screen the genes with significant differences among the different treatments. Differently expressed genes were calculated using DESeq2 v1.39.0 software with significance thresholds of Adjust *p* < 0.05 and |log_2_FoldChange| > 2, where *p* values were corrected using the BH method. Furthermore, as for the analysis of flow apoptosis graphs and the conjugation transfer experiment in the complex multi-microbial system, the Flowjo software (v10.8.1) was used to analyze and plot those results. And the sequence result images of the plasmids were obtained using Snap Gene (v6.0.2).

**REFERENCES**

1. Zhang, Shuai, Yue Wang, Hailiang Song, Ji Lu, Zhiguo Yuan, Jianhua Guo. 2019. “Copper nanoparticles and copper ions promote horizontal transfer of plasmid-mediated multi-antibiotic resistance genes across bacterial genera.” *Environment International* 129: 478-487. https://doi.org/10.1016/j.envint.2019.05.054

2. Park, Geunyung, Jina Yang, Sang Woo Seo. 2024. “Dynamic control of the plasmid copy number maintained without antibiotics in Escherichia coli.” *Journal of Biological Engineering* 18: 71. <https://doi.org/10.1186/s13036-024-00460-1>

3. Oftadeh, Omid, Vassily Hatzimanikatis. 2024. “Genome-scale models of metabolism and expression predict the metabolic burden of recombinant protein expression.” *Metabolic Engineering* 84: 109-116. <https://doi.org/10.1016/j.ymben.2024.06.005>

4. Yuan, Qingbin, Ruonan Sun, Pingfeng Yu, Yuan Cheng, Wenbin Wu, Jiming Bao, Pedro J. J. Alvarez. 2022. “UV-aging of microplastics increases proximal ARG donor-recipient adsorption and leaching of chemicals that synergistically enhance antibiotic resistance propagation.” *Journal of Hazardous Materials* 427: 127895. [https://doi.org/10.1016/j.jhazmat.2021.127895](https://doi.org/https://doi.org/10.1016/j.jhazmat.2021.127895)

5. Chávez-Calderón, Adriana, Francisco Paraguay-Delgado, Erasmo Orrantia-Borunda, Antonia Luna-Velasco. 2016. “Size effect of SnO2 nanoparticles on bacteria toxicity and their membrane damage.” *Chemosphere* 165: 33-40. [https://doi.org/10.1016/j.chemosphere.2016.09.003](https://doi.org/https://doi.org/10.1016/j.chemosphere.2016.09.003)

**Figure S1 Drug sensitive test of *E. coli* K12 exposure to NPs with different charge under low (5 mg/L) or high (50 mg/L) concentration.** Here are drug sensitive tests for AMP, KA and NOR antibiotics. Total of five treatments, including a control without nanoplastics (NPs) (CK), NPs with PC under low (L-PC) and high concentration (H-PC), and NPs with NC under low concentration (L-NC) and high concentration (H-NC). The grey, green, and orange lines represent the CK, PC, and NC treatment, respectively.

**Figure S2** **Distribution of the relative abundance of Fluoroquinolones and β-lactam ARGs under different treatments.** The grey, green, and orange lines represent the CK, PC, and NC treatment, respectively. And the white squares represent average relative abundance of ARGs. “ns” indicates no significant difference, with *p* > 0.05. “*” represents a significant difference, with *p* < 0.05.

**
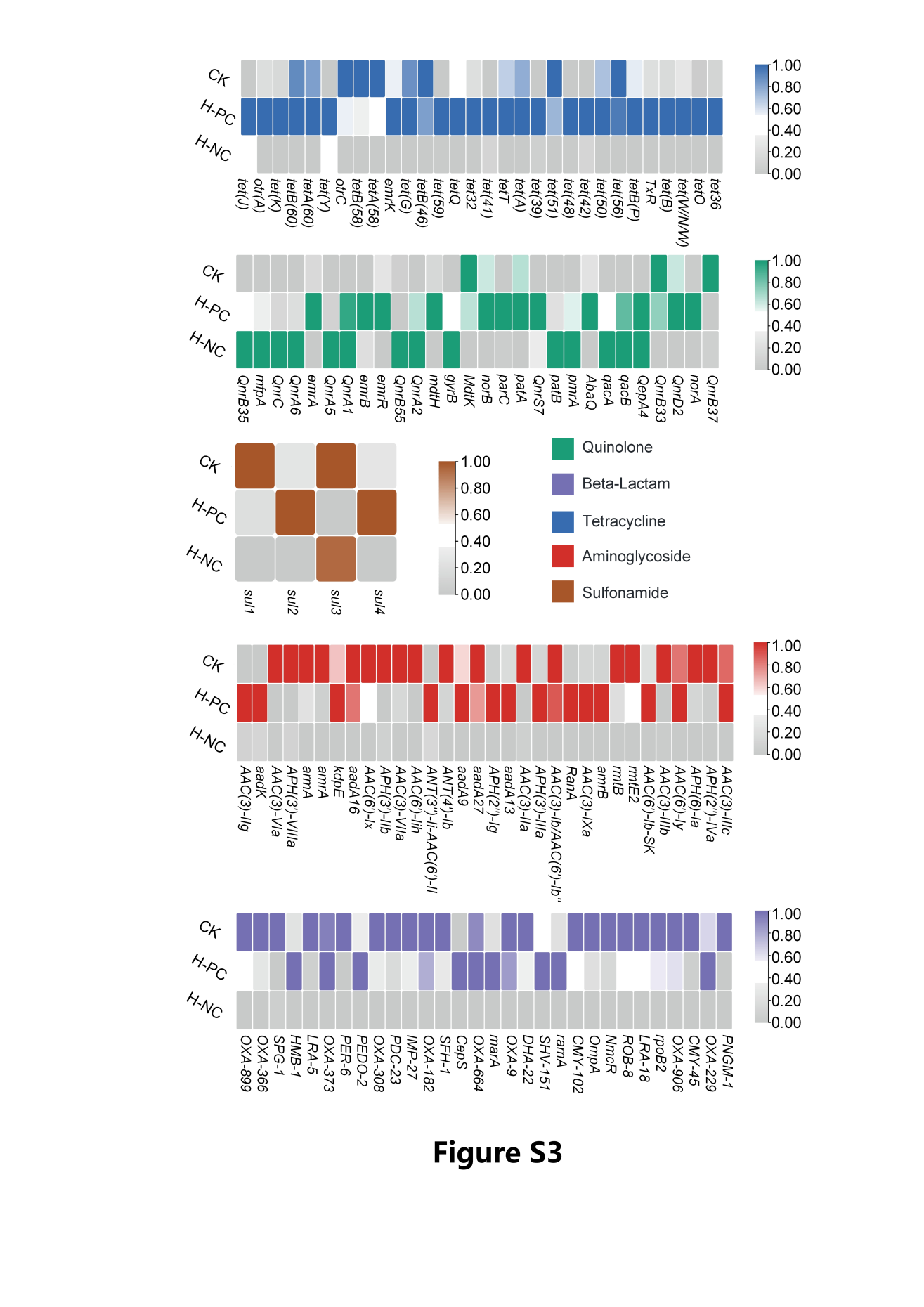
**

**Figure S3** **Heatmap shows that the relative abundance of ARG subtypes under different treatments.** The data are normalized from 0 to 1. The green, purple, blue, red, and brown represent the quinolone, Beta-lactam, tetracyclinde, aminoglycoside, and sulfonamide resistance genes, respectively.

**
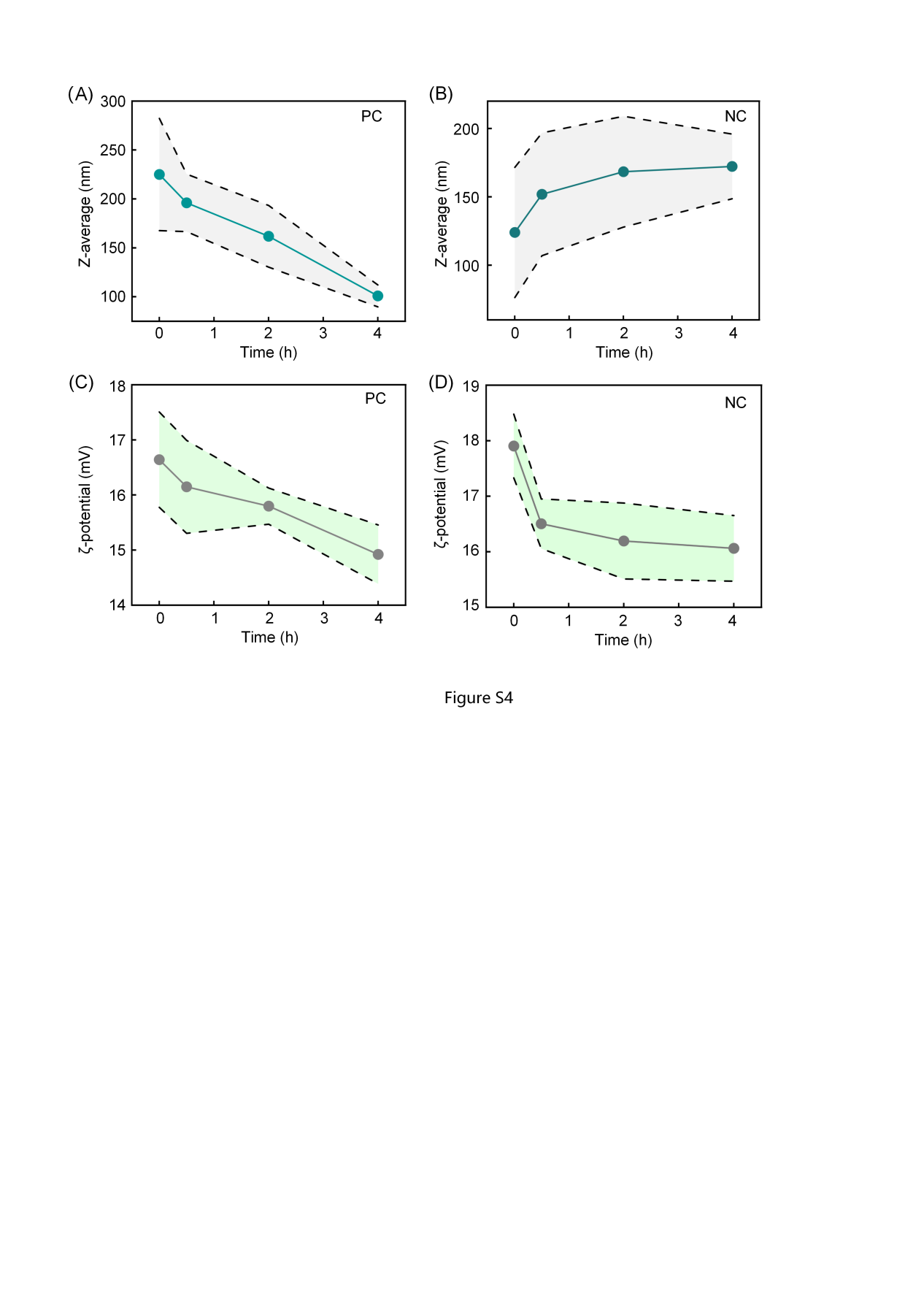
**

**Figure S4 Z-average hydrodynamic diameter and ζ-Potential of NPs in LB.** (A−B) The Z-average hydrodynamic diameter of NPs under NC-PC and NP-NC treatments in LB. (C−D) The ζ-Potential of NPs under NC-PC and NP-NC treatments in LB.

**
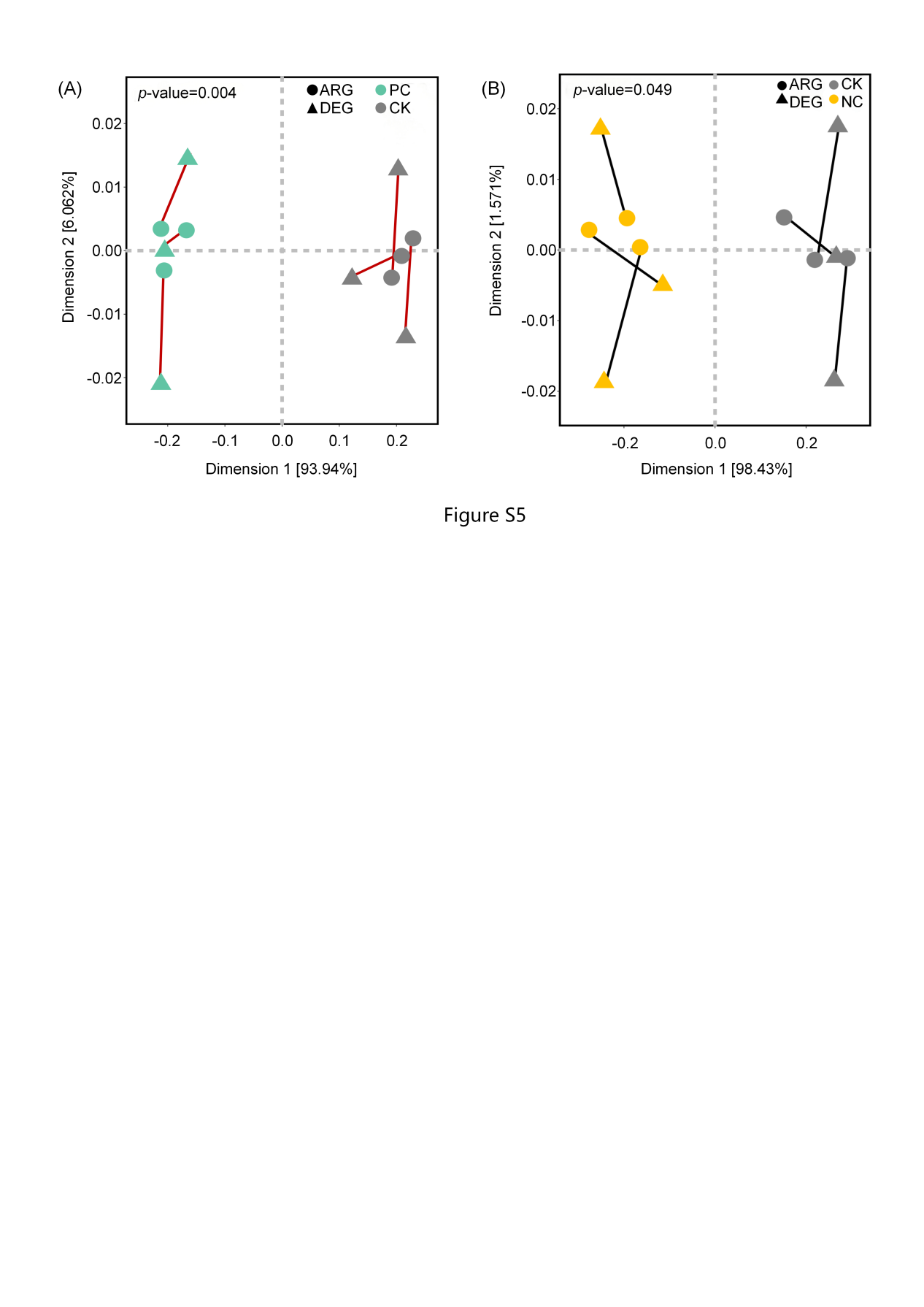
**

**Figure S5 Procrustes analysis displays the relationships between DEGs and ARGs under different treatments.** (A) The relationships between ARGs and DEGs under NP-PC and CK treatments. (B) The relationships between ARGs and DEGs under NP-NC and CK treatments.

**
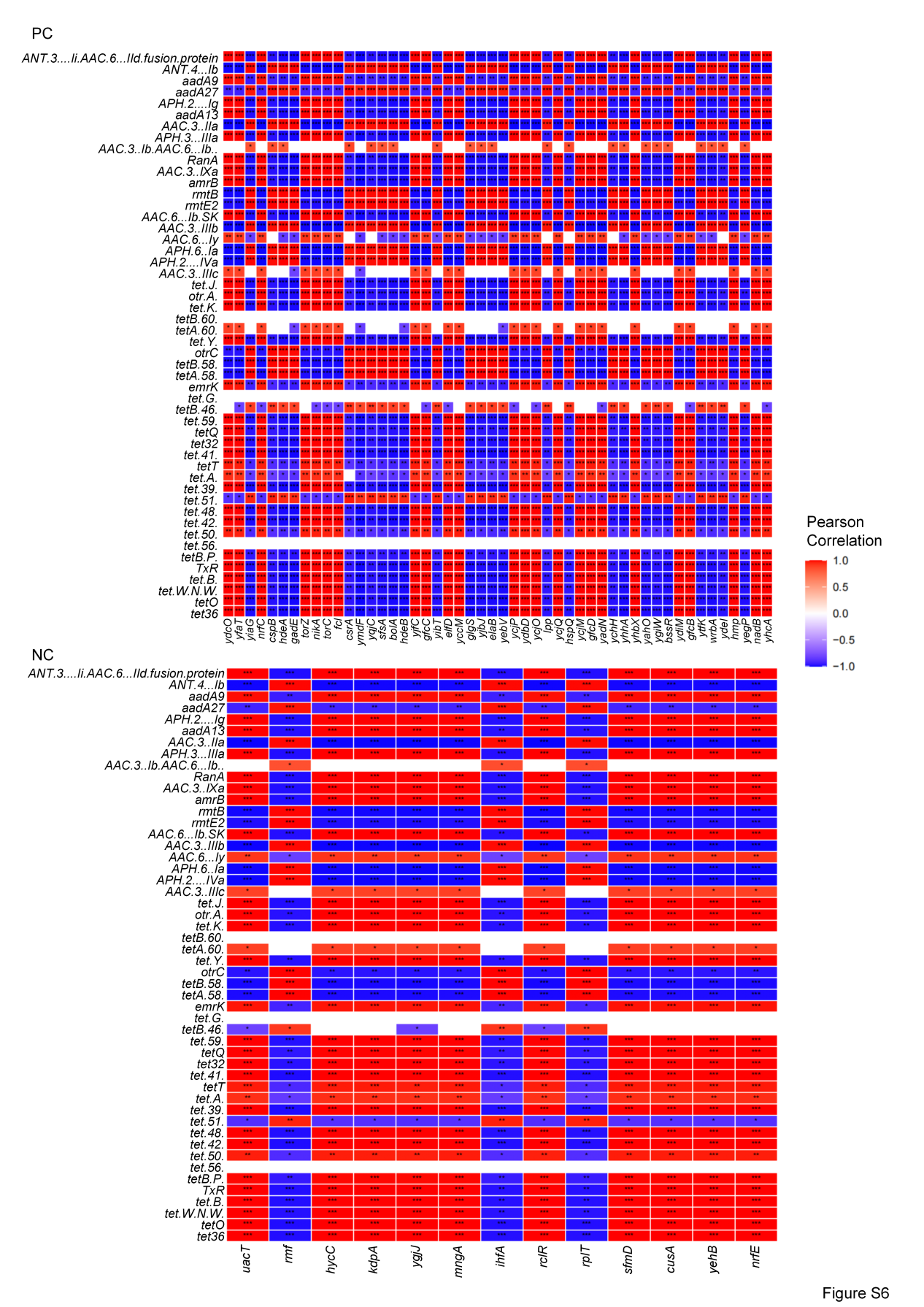
**

**Figure S6 Heatmap shows the relationships between DEGs and ARGs.** Red represents positive correlations and blue represents negative correlations. “*” represents *p* < 0.05, “**” represents *p* < 0.01, and “***” represents *p* < 0.001.

**Figure S7 Expression of DEGs under different treatments.** (A) CK *vs.* PC. (B) CK *vs.* NC.

**Figure S8 Proteomic analysis of *E. coli* under exposure to NPs with different surface charges.** (A) Number of DEPs (|Log_2_FoldChange| > 2) in the NP-PC and NP-NC treatments compared with CK treatment. (B) Number of up-regulation and down-regulation DEPs under different treatments. (C) Enrichment analysis of DEPs based on the GO database. “*” represents a significant difference, with *p* < 0.05.

**Figure S9 Whole genome sequencing (WGS) and the number and length of plasmid carried by the *E. coli.***


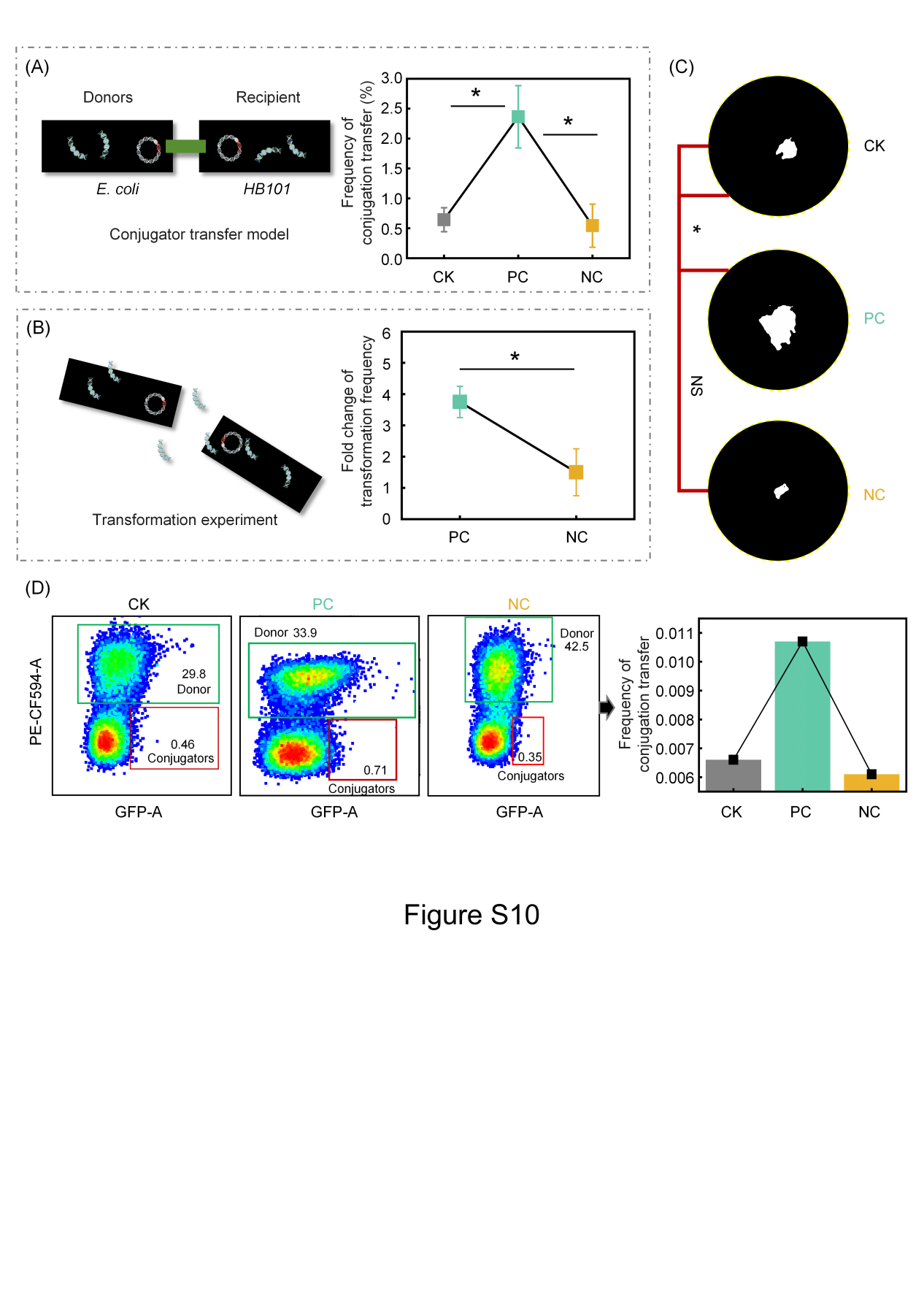


**Figure S10 Horizontal gene transfer ability under different treatments.** (A) Conjugation transfer frequency of *E. coli*. (B) Transformation ability of free plasmid for *E. coli*. (C) Swarming test: the crawl ability of *E. coil*. (D) Horizontal gene transfer ability of plasmids carried by *E. coli* in a multi-bacterial system by flow cytometry. “*” represents a significant difference, with *p* < 0.05.

**
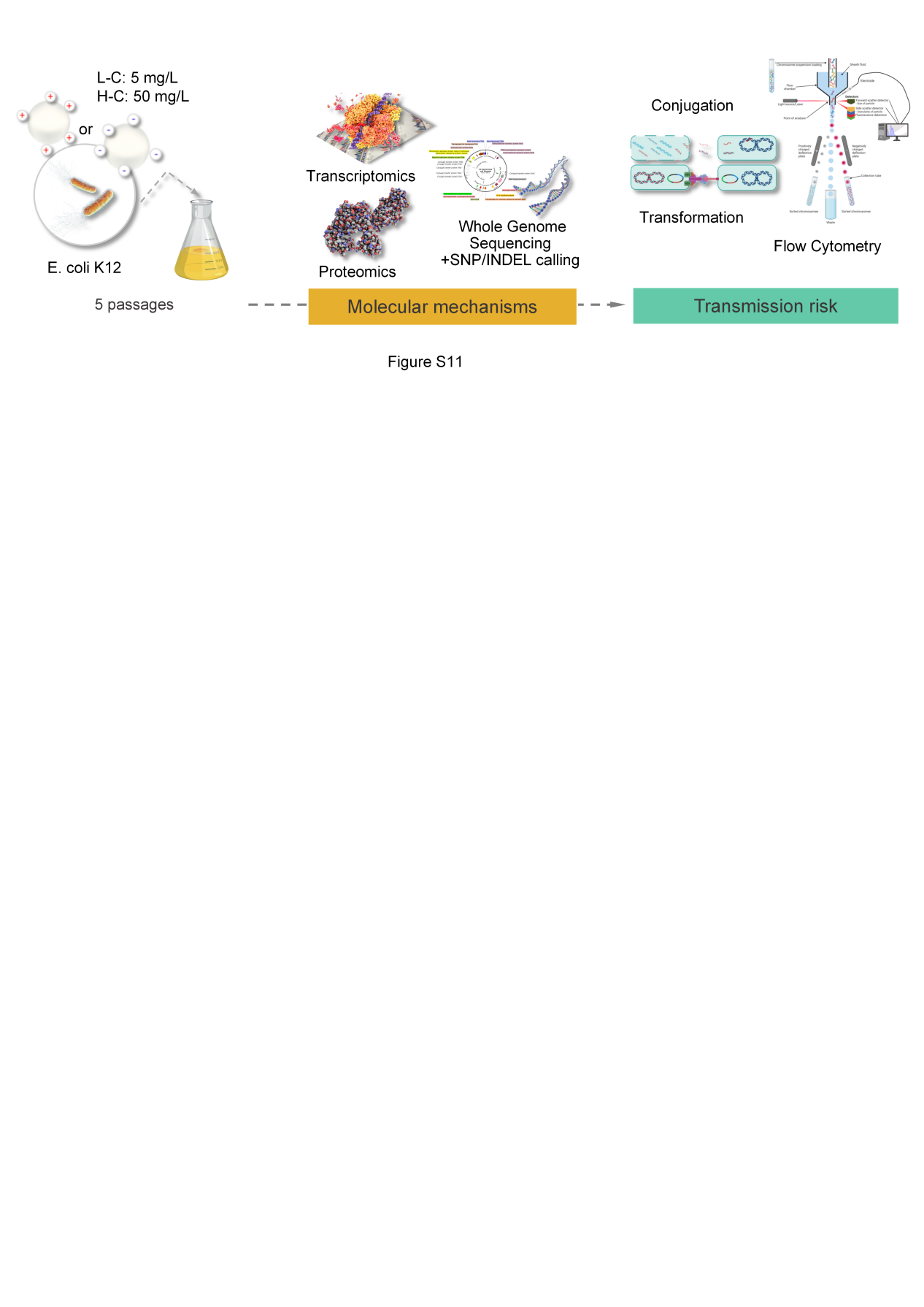
**

**Figure S11 Environmental design diagram.**


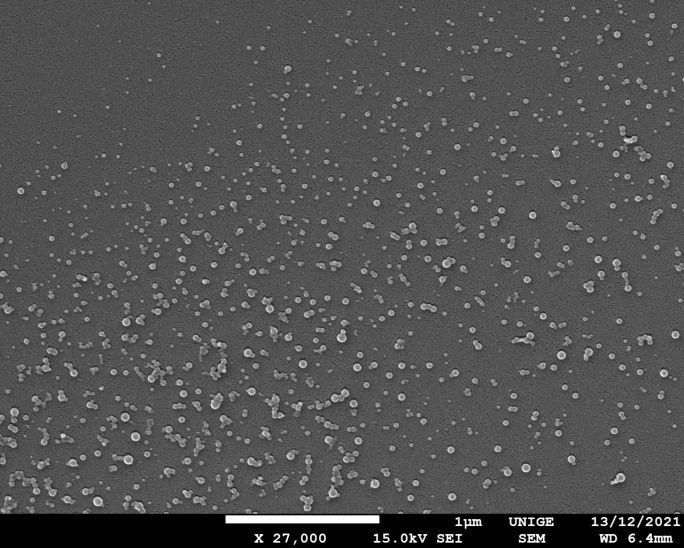


**Figure S12 Morphology of NPs (20 nm) is examined using SEM.** NPs are shaken to disperse after ultrasonic oscillationing and are imaged at the 1 μm scale.
